# Pick Your Poison Carefully: How Alcohol Consumption and Serum Biomarkers Influence Body Fat – A UK Biobank Study

**DOI:** 10.1093/geroni/igaa057.3280

**Published:** 2020-12-16

**Authors:** Brittany Larsen, Brandon Klinedinst, Scott Le, Colleen Pappas, Nathan Meier, Ye-Lim Lim, Tovah Wolf, Auriel Willette

**Affiliations:** 1 Iowa State University, Ames, Iowa, United States; 2 Concordia University Irvine, Irvine, California, United States; 3 Virginia Polytechnic Institute, Blacksburg, Virginia, United States; 4 Western Carolina University, Cullowhee, North Carolina, United States

## Abstract

Aging is characterized by physiological alterations in body composition, such as increased visceral adiposity accumulation and bone loss. Alcohol consumption is thought to partially drive these associations, but findings have been mixed. To clarify inconsistent findings, different types of alcohol--beer, liquor, and wine--may show different association patterns with body composition. Our longitudinal U.K. Biobank study leveraged 1,874 White British participants (aged 40-79 years; 58.9% male). Participants self-reported demographic, alcohol and dietary consumption patterns, and lifestyle factors using a touchscreen questionnaire. Anthropometrics and serum for proteomics were collected and body composition was obtained via dual-energy X-ray absorptiometry (DEXA). Structural equation modeling was used to probe direct and indirect associations between adiposity and bone, alcohol types, and cardiometabolic biomarkers. Over a mean duration of four years, greater consumption of beer and liquor were significantly associated with more visceral adiposity (β=.069, p<.001 and β=.014, p<.001, respectively); these associations were driven by dyslipidemia and insulin resistance. In contrast, greater red wine consumption predicted less adipose mass (β=-.023, p<.001), and this association was mediated by reduced inflammation and higher high-density lipoproteins (HDL) cholesterol. White wine consumption did not influence visceral adiposity but did predict greater bone mineral density (BMD) (β=.051, p=.003). Taken together, these data suggest that beer and liquor may drive the “empty calorie” hypothesis related to adipogenesis, while red wine may be protective due to anti-inflammatory and eulipidemic effects. Furthermore, white wine may benefit bone mineral density in older adults.

